# Rapid identification of methylase specificity (RIMS-seq) jointly identifies methylated motifs and generates shotgun sequencing of bacterial genomes

**DOI:** 10.1093/nar/gkab705

**Published:** 2021-08-20

**Authors:** Chloé Baum, Yu-Cheng Lin, Alexey Fomenkov, Brian P Anton, Lixin Chen, Bo Yan, Thomas C Evans, Richard J Roberts, Andrew C Tolonen, Laurence Ettwiller

**Affiliations:** New England Biolabs, Inc. 240 County Road Ipswich, MA 01938, USA; Génomique Métabolique, Genoscope, Institut François Jacob, CEA, CNRS, Univ Evry, Université Paris-Saclay, 91000 Évry, France; New England Biolabs, Inc. 240 County Road Ipswich, MA 01938, USA; New England Biolabs, Inc. 240 County Road Ipswich, MA 01938, USA; New England Biolabs, Inc. 240 County Road Ipswich, MA 01938, USA; New England Biolabs, Inc. 240 County Road Ipswich, MA 01938, USA; New England Biolabs, Inc. 240 County Road Ipswich, MA 01938, USA; New England Biolabs, Inc. 240 County Road Ipswich, MA 01938, USA; New England Biolabs, Inc. 240 County Road Ipswich, MA 01938, USA; Génomique Métabolique, Genoscope, Institut François Jacob, CEA, CNRS, Univ Evry, Université Paris-Saclay, 91000 Évry, France; New England Biolabs, Inc. 240 County Road Ipswich, MA 01938, USA

## Abstract

DNA methylation is widespread amongst eukaryotes and prokaryotes to modulate gene expression and confer viral resistance. 5-Methylcytosine (m5C) methylation has been described in genomes of a large fraction of bacterial species as part of restriction-modification systems, each composed of a methyltransferase and cognate restriction enzyme. Methylases are site-specific and target sequences vary across organisms. High-throughput methods, such as bisulfite-sequencing can identify m5C at base resolution but require specialized library preparations and single molecule, real-time (SMRT) sequencing usually misses m5C. Here, we present a new method called RIMS-seq (rapid identification of methylase specificity) to simultaneously sequence bacterial genomes and determine m5C methylase specificities using a simple experimental protocol that closely resembles the DNA-seq protocol for Illumina. Importantly, the resulting sequencing quality is identical to DNA-seq, enabling RIMS-seq to substitute standard sequencing of bacterial genomes. Applied to bacteria and synthetic mixed communities, RIMS-seq reveals new methylase specificities, supporting routine study of m5C methylation while sequencing new genomes.

## INTRODUCTION

DNA modifications catalysed by DNA methyltransferases are considered to be the most abundant form of epigenetic modification in genomes of both prokaryotes and eukaryotes. In prokaryotes, DNA methylation has been mainly described as part of the sequence-specific restriction modification system (RM), a bacterial immune system to resist invasion of foreign DNA (1). As such, profiling methylation patterns gives insight into the selective pressures driving evolution of their genomes.

Around 90% of bacterial genomes contain at least one of the three common forms of DNA methylation: 5-methylcytosine (m5C), N4-methylcytosine (m4C) and N6-methyladenine (m6A)) (2,3). Contrary to eukaryotes where the position of the m5C methylation is variable and subject to epigenetic states, bacterial methylations tend to be constitutively present at specific sites across the genome. These sites are defined by the methylase specificity and, in the case of RM systems, tend to be fully methylated to avoid cuts by the cognate restriction enzyme. The methylase recognition specificities typically vary from four to eight nucleotides and are often, but not always, palindromic (4).

PacBio single molecule, real-time (SMRT) sequencing has been instrumental in the identification of methylase specificity largely because, in addition to providing long read sequencing of bacterial genomes, m6A and m4C can easily be detected using the characteristic interpulse duration (IPD) of those modified bases (5). Thus, a single run on PacBio allows for both the sequencing and assembly of unknown bacterial genomes and the determination of m6A and m4C methylase specificities. However, because the signal associated with m5C bases is weaker than for m6A or m4C, the IPD ratio of m5C is very similar to the IPD of unmodified cytosine. Thus, PacBio sequencing misses the m5C methylase activities (2) unless the 5-methylcytosine detection is enhanced by treating the library with Ten-eleven translocation enzyme (6). A recent study uses a holistic kinetic model to identify m5C using PacBio reads (7). Nonetheless, methylation can only be identified in CpG context, restricting the use of this approach to organisms such as human, for which methylation is almost exclusively in CpG sites.

Consequently, the identification of m5C requires specialized methods such as bisulfite sequencing, enzyme-based techniques such as EM-seq (8) or hybrid techniques such as TAPS-seq (9). Recently, MFRE-Seq has been developed to identify m5C methylase specificities in bacteria (10). MFRE-Seq uses a modification-dependent endonuclease that generates a double-stranded DNA break at methylated sites, allowing the identification of m5C for the subset of sites conforming to the recognition sites of the MFRE enzymes. Unlike PacBio sequencing, these specialized methods do not provide the dual original sequence and methylation readouts from a single experiment.

Recently, m5C in the CpG context has been identified (11) and a signal for methylation can be observed at known methylated sites in bacteria using Nanopore sequencing (12,13). So far no technique permits, from a single experiment, the dual sequencing of genomes and the *de novo* determination of m5C methylase specificity for the non-CpG contexts typically found in bacteria.

Herein, we describe a novel approach called RIMS-seq to simultaneously sequence bacterial genomes and globally profile m5C methylase specificity using a protocol that closely resembles the standard Illumina DNA-seq with a single, additional step. RIMS-seq shows comparable sequencing quality as DNA-seq and accurately identifies methylase specificities. Applied to characterized strains or novel isolates, RIMS-seq *de novo* identifies novel activities without the need for a reference genome and permits the assembly of the bacterial genome at metrics comparable to standard shotgun sequencing.

## MATERIALS AND METHODS

### Samples and genomic DNA collection

Skin microbiome genomic DNA (ATCC^®^ MSA-1005) and gut microbiome genomic DNA (ATCC^®^ MSA-1006) were obtained from ATCC. *Escherichia coli* BL21 genomic DNA was extracted from a culture of *E. coli* BL21 DE3 cells (C2527, New England Biolabs) using the DNEasy Blood and Tissue kit (69504, Qiagen). *Escherichia coli* K12 MG1655 genomic DNA was extracted from a cell culture using the DNEasy Blood and Tissue kit (69504, Qiagen). All the other gDNA from the bacteria presented in Table 1 were isolated using the Monarch genomic DNA purification kit (T3010S, New England Biolabs). Xp12 phage genomic DNA was obtained from Peter Weigele and Yian-Jiun Lee at New England Biolabs.

**Table 1.** Methylases specificity obtained using RIMS-seq and validated using different methods. The method is indicated by a number next to the motif. : Evidence for the validated motifs are (1) bisulfite-seq (Materials and Methods), (2) REBASE (4), (3) EM-seq (material and method), (4) MFRE-seq (10), (5) mTet1-enhanced SMRT sequencing (6)

Organism	Accession numbers (biosample)	RIMS-seq motif(s)	Validated motif(s)
*Escherichia coli* K12	SAMN02604091	CCWGG	CCWGG (1,2,4)
*Acinetobacter calcoaceticus* ATCC 49823	SAMN14530202	GATC	GATC (4)
		CGCG	CGCG (2,4)
*Bacillus fusiformis* 1083	SAMN17843035	ACCTGC	ACCTGC (2,3)
		GCAGGT	GCAGGT (2,3)
*Bacillus amyloliquefaciens* H ATCC 49763	SAMN12284742	GCWGC	GCWGC (3)
*Clostridium acetobutylicum* ABKn8	SAMN17843114	GCNNGC	GCNNGC (3)
*Aeromonas hydrophila* NEB724	SAMN14533640	GCCGGC	GCCGGC (3)
*Haemophilus influenzae* Rd ATCC 51907	SAMN02603991	GRCGYC*	GRCGYC (5)
		ACCGCACT	
		AGTGCGGT	
*Haemophilus parahaemoltyicus* ATCC 10014	SAMN11345835	GCGC	GCGC (2)
M.HhaI clone (*E. coli*)	NA	RCGC	GCGC (4)
		CCWGG^(a)^	CCWGG (1,2,4)^(a)^

(a) The *E. coli* strain used is Dcm+, resulting in the discovery of both the Dcm (CCWGG) and M.HhaI motifs (GCGC). RIMS-seq discovered RCGC instead of GCGC motif (see text for explanation). * *P*-value = 1.0e^−91^ (standard detection threshold of <1.0e^−100^ would miss this motif).

### RIMS-seq library preparation

One hundred nanogram of gDNA was sonicated in 1× TE buffer using the Covaris S2 (Covaris) with the standard protocol for 50 μl and 200 bp insert size.

The subsequent fragmented gDNA was used as the starting input for the NEBNext Ultra II library prep kit for Illumina (E7645, New England Biolabs) following the manufacturer's recommendations until the USER treatment step. The regular unmethylated loop-shaped adapter was used for ligation. After the USER treatment (step included), the samples were subjected to heat alkaline deamination: 1 M NaOH pH 13 was added to a final concentration of 0.1 M and the reactions were placed in a thermocycler at 60°C for 3 h. Then, the samples were immediately cooled down on ice and 1 M of acetic acid was added to a final concentration of 0.1 M in order to neutralize the reactions. We also tested alkaline concentration of 0.5M and 1M NaOH, in these cases, equal amounts of acetic acid were added to the reaction to properly neutralize the PH. The neutralized reactions were cleaned up using the Zymo oligo clean and concentrator kit (D4060 Zymo Research) and the DNA was eluted in 20 μl of 0.1× TE.

PCR amplification of the samples was done following NEBNext Ultra II library prep kit for Illumina protocol (ER7645, New England Biolabs) and the NEBNext^®^ Multiplex Oligos for Illumina^®^ (E7337A, New England Biolabs). The number of PCR cycles was tested and optimized for each sample following the standard procedure for library preparation. PCR reactions were cleaned up using 0.9× NEBNext Sample purification beads (E7137AA, New England Biolabs) and eluted in 25 μl of 0.1× TE. All the libraries were evaluated on a TapeStation High sensitivity DNA1000 (Agilent Technologies) and paired-end sequenced on Illumina.

### Bisulfite-seq library preparation

One percent of lambda phage gDNA (D1221, Promega) was spiked-into 300 ng gDNA to use as an unmethylated internal control. The samples were sonicated in 1× TE buffer using the Covaris S2 (Covaris) with the standard protocol for 50 μl and 200 bp insert size.

The subsequent fragmented gDNA was used as the starting input for the NEBNext Ultra II library prep kit for Illumina (E7645, New England Biolabs) following the manufacturer's recommendations until the USER treatment step. The methylated loop-shaped adapter was used for ligation. After USER, a 0.6× clean-up was performed using the NEBNext Sample purification beads (E7137AA, New England Biolabs) and eluted in 20 μl of 0.1× TE. A TapeStation High Sensitivity DNA1000 was used to assess the quality of the library before subsequent bisulfite treatment. The Zymo EZ DNA Methylation-Gold Kit (D5005, Zymo Research) was used for bisulfite treatment, following the manufacturer's protocol.

PCR amplification of the samples was done following the suggestions from NEBNext Ultra II library prep kit for Illumina (ER7645, New England Biolabs), using the NEBNext^®^ Multiplex Oligos for Illumina^®^ (E7337A, New England Biolabs) and NEBNext^®^ Q5U^®^ Master Mix (M0597, New England Biolabs).

The number of PCR cycles was tested and optimized for each sample. The PCR reactions were cleaned up using 0.9× NEBNext Sample purification beads (E7137AA, New England Biolabs) and eluted in 25 μl of 0.1× TE. All the libraries were screened on a TapeStation High sensitivity DNA1000 (Agilent Technologies) and paired-end sequenced on Illumina.

### RIMS-seq data analysis

Paired-end reads were trimmed using Trim Galore 0.6.3 (option –trim1). The *Acinetobacter calcoaceticus* ATCC 49823 data have been trimmed using Trim Galore version 0.6.3 instead and downsampled to 1 million reads. Reads were mapped to the appropriate genome using BWA mem with the paired-end mode (version 0.7.5a-r418 and version 0.7.17-r1188 for the *A. calcoaceticus*). When using an assembled genome directly from RIMS-seq data, trimmed RIMS-seq reads were assembled using SPAdes (SPAdes-3.13.0 (31) default parameters). Reads were split according to the read origin (Read 1 or Read 2) using samtools (version 1.8) with -f 64 (for Read 1) and -f 128 (for Read 2) and samtools mpileup (version 1.8) was run on the split read files with the following parameters: -O -s -q 10 -Q 0. For *Acinetobacter calcoaceticus*, the unmapped reads, reads without a mapped mate and the non-primary alignments were filtered out using the flags -F 12 and -F 256.

### *De-novo* identification of motifs using RIMS-seq

Programs and a detailed manual for the *de-novo* identification of motifs in RIMS-seq are available on github (https://github.com/Ettwiller/RIMS-seq/). Using the mpileup files, positions and 14bp flanking genomic regions for which a high quality (base quality score ≥ 35) C to T in R1 or G to A in R2 was found, were extracted for the foreground. Positions and 14bp flanking regions for which a high quality (base quality score ≥ 35) G to A in R1 or C to T in R2 was found, were extracted for the background. C to T or G to A in the first position of reads were ignored. If the percentage of C to T or G to A are above 5% for at least 5 reads at any given position, the position was ignored (to avoid considering positions containing true variants). Motifs that are found significantly enriched (*P*-value < 1e^−100^) in the foreground sequences compared to background sequences were found using mosdi pipeline mosdi-discovery with the following parameters: ‘*mosdi-discovery -v discovery -q x -i -T 1e-100 -M 8,1,0,4 8 occ-count’* using the foreground sequences with *x* being the output of the following command : ‘*mosdi-utils count-qgrams -A ‘dna’*’ using the background sequences. To identify additional motifs, the most significant motif found using *mosdi-discovery* is removed from the foreground and background sequences using the following parameter: ‘*mosdi-utils cut-out-motif -M X’* and the motif discovery process is repeated until no significantly enriched motif can be found.

### Sequence logo generation

Using the mpileup files, positions in the genome for which a high quality (base quality score ≥ 35) C to T in R1 or a G to A in R2 was observed were extracted for the foreground using the get_motif_step1.pl program. Positions for which a high quality (base quality score ≥ 35) G to A in R1 or a C to T in R2 was observed were extracted for the background. The ±7 bp regions flanking those positions were used to run two sample logo (32). Parameters were set as *t*-test, *pP*-value <0.01.

### Bisulfite-seq data analysis

Reads were trimmed using Trim Galore 0.6.3 and mapped to the bisulfite-converted concatenated reference genomes of each respective synthetic microbiome using bismark 0.22.2 with default parameters. PCR duplicates were removed using deduplicate_bismark and methylation information extracted using bismark_methylation_extractor using default parameters. For the microbiome, the bismark_methylation_extractor with –split_by_chromosome option was used to output one methylation report per bacterium. The motif identification was done as previously described in (10).

### EM-seq

EM-seq was performed according to the standard protocol (NEB E7120S). Motif identification was done as previously described in (10).

### Analysis and abundance estimation in synthetic microbiomes

RIMS-seq, DNA-seq and bisulfite-seq were performed on the synthetic gut and skin microbiome as described. Reads derived from RIMS-seq, DNA-seq and bisulfite-seq were mapped as described to a ‘meta-genome’ composed of the reference genomes of all the bacteria included in the corresponding synthetic community (see Supplementary Table S3 for detailed compositions). Mapped reads were split according to each bacterium and RIMS-seq or bisulfite analysis pipelines were run on individual genomes as described above. Abundance was estimated using the number of mapped reads per bacteria and normalized to the total number of mapped reads. Normalized species abundances in RIMS-seq and bisulfite-seq were compared to the normalized species abundances in DNA-seq.

### Phylogeny of the ATCC synthetic microbiomes and visualization

The phylogenetic trees of both ATCC synthetic gut and skin microbiomes were done using OrthoFinder version 2.3.11 (33) using the MSA workflow and MAFFT for the multiple sequence alignment program. The program options are available at https://github.com/davidemms/OrthoFinder. The phylogenetic tree and abundance data obtained from DNA-seq, RIMS-seq and bisulfite-seq were visualized using iTOL (34) for each synthetic community (see Supplementary Figure S5).

### Quality control of the data

The insert size for each downsampled filtered bam file was calculated using Picard version 2.20.8 using the default parameters and the option CollectInsertSizeMetrics (‘Picard Toolkit.’ 2019. Broad Institute, GitHub Repository. http://broadinstitute.github.io/picard/; Broad Institute).

The GC bias for each downsampled filtered bam file was calculated and plotted using Picard version 2.20.8 using the default parameters and the option CollectGcBiasMetrics.

### Xp12 genome assembly

Reads were downsampled to a 30× coverage using seqtk 1.3.106, trimmed using trimgalore 0.6.5 and assembled using Spades 3.14.1 with the –isolate option. Assembly quality was assessed using Quast 5.0.2. Reads used for assembly were then mapped back to the assembly using BWA mem 0.7.17 and mapping statistics were generated using samtools flagstat 1.10.2

### Xp12 sequencing performance assessment

Reads were trimmed using trimgalore 0.6.5 and mapped to the Xp12 reference genome using BWA mem 0.7.17. Insert size and GC bias were assessed using Picard Toolkit and genome coverage using Qualimap 2.1.1.

### Intact mass LC–MS

Intact mass analysis was performed by tandem liquid chromatography–mass spectrometry (LC–MS/MS) on an Vanquish Horizon UHPLC System equipped with a diode array detector and a Thermo Q-Exactive Plus mass spectrometer operating under negative electrospray ionization mode (–ESI). UHPLC was performed using a Thermo DNAPac™ RP Column (2.1 × 50 mm, 4 μm) at 70°C and 0.3 ml/min flow rate, with a gradient mobile phase consisting of hexafluoroisopropanol (HFIP)–*N*,*N*-diisopropylethylamine (DIEA) aqueous buffer and methanol. UV detection was performed at 260 nm. Intact mass analysis was performed under Full MS mode, and ESI-MS raw data was deconvoluted using Promass HR (Novatia Inc.).

## RESULTS

### Principle of RIMS-seq

**Figure 1.**
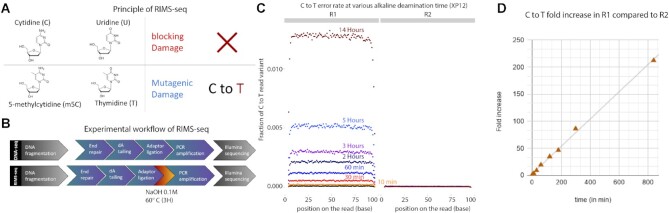
(**A**) Principle of RIMS-seq. Deamination of cytidine leads to a blocking damage while deamination of m5C leads to a mutagenic C to T damage only present on the first read (R1) of paired-end reads in standard Illumina sequencing. Thus, an increase of C to T errors in R1 in specific contexts is indicative of m5C. (**B**) The workflow of RIMS-seq is equivalent to a regular library preparation for Illumina DNA-seq with an extra step of limited alkaline deamination at 60°C. This step can be done immediately after adaptor ligation and does not require additional cleaning steps. (**C**) Fraction of C to T variants in XP12 (m5C) at all positions in the reads for R1 and R2 after 0min (DNA-seq), 10 min, 30 min, 60 min, 2 h, 3 h, 5 h and 14 h of heat-alkaline treatment. The C to T imbalance between R1 and R2 is indicative of deamination of m5C and increases with heat-alkaline treatment time. (**D**) Correlation between the C to T fold increases in R1 compared to R2 according to time (*r*^2^= 0.998).

Spontaneous deamination of cytosine (C) leading to uracil (U) and of m5C leading to thymine (T) are examples of common damage found in DNA. *In-vitro*, this damage is often undesirable as it can interfere with sequencing. The type of interference during sequencing depends on whether the deamination occurs on C or m5C. U blocks the passage of high-fidelity polymerases typically used in library preparation protocols, preventing the amplification and sequencing of U-containing DNA fragments. Conversely, DNA harboring T derived from m5C deamination can be normally amplified but results in C to T errors (14,15). This distinction between blocking and mutagenic damage forms the basis of the RIMS-seq method, allowing the identification of methylase specificity based on an elevated number of reads containing C to T transitions specifically at methylated sites (Figure 1A). To increase the rate of m5C deamination, the DNA is subjected to a heat-alkaline treatment which has been previously demonstrated to elevate the rate of both C and m5C deamination with m5C having a 1.5–3 times higher deamination rate than for C (16). This treatment is aimed at inducing a level of deamination large enough to detect the m5C methylase specificity without affecting the sequencing quality. For this reason, the deamination levels typically obtained with RIMS-seq does not permit the quantitative measurement of methylation at each genomic site but rather provides a global methylation signal characteristic of the methylase specificity.

Illumina paired-end sequencing allows both ends of a DNA fragment to be sequenced, generating a forward read (R1) and reverse read (R2). Resulting from m5C deamination, R1 has the C to T read variants while R2 has the reverse-complement G to A variant. This difference leads to an overall imbalance of C to T variants between R1 and R2 (17) (see also Supplementary Figure S1 for explanation). Thus, sequence contexts for which the C to T read variants are imbalanced in R1 compared to R2 correspond to m5C methylase specificity(ies). Because of the limited deamination rate, RIMS-seq takes advantage of the collective signal at all sites to define methylase specificity. Because C to T imbalance can be observed at nucleotide resolution, RIMS-seq identifies at base resolution which of the cytosine within the motif is methylated.

The experimental steps for RIMS-seq essentially follow the standard library preparation for Illumina sequencing with an extra deamination step. Briefly, the bacterial genomic DNA is fragmented, and adaptors are ligated to the ends of DNA fragments (Figure 1B and Materials and Methods). Between the ligation step and the amplification step, an alkaline heat treatment step is added to increase the rate of deamination. Only un-deaminated DNA or DNA containing deaminated m5C can be amplified and sequenced.

### Validation of RIMS-seq

#### Optimization of the heat alkaline deamination step

We first evaluated the conditions to maximize the deamination of m5C while minimizing other DNA damage. For this we used bacteriophage Xp12 genomic DNA that contains exclusively m5C instead of C (18) to measure the m5C deamination rates in various contexts.

To estimate the overall deamination rate of m5C, we quantified the imbalance of C to T read variants between R1 and R2 for 0, 10 and 30 min, 1 h, 2 h, 3 h, 5 h and 14 h of heat alkaline treatment (Figure 1C). We observed an imbalance as early as 10 min with a 3.7-fold increase of C to T read variants in R1 compared to R2. The increase is linear with time with a maximum of 212-fold increase of C to T read variants in R1 compared to R2 after 14 h of heat alkaline treatment (Figure 1D). Next, we quantified the deamination rate at all Nm5CN sequence contexts with N being A, T, C or G and show an increase of C to T variants in R1 in all contexts (Supplementary Figure S2A). Together, these results show that a measurable deamination rate can be achieved in as soon as 10 min of heat alkaline deamination and that deamination efficiency is similar in all sequence contexts.

To estimate the non-specific damage to the DNA leading to unwanted sequencing errors, we quantified possible imbalances for other variant types (Supplementary Figure S2B). We found that G to T variants show imbalance in all the conditions investigated, likely the result of oxidative damage resulting from sonication, a common step in library preparation between RIMS-seq and DNA-seq (17). Interestingly, the imbalance is reduced in RIMS-seq, disappearing almost completely after 14 h of heat alkaline treatment (Supplementary Figure S2B). This result suggests that this treatment either converts 8-oxoG back to G or to another modification that ultimately blocks the polymerase from amplifying 8-oxoG-containing fragments. To properly address the disappearance of G to T variants due to oxidative damage in RIMS-seq, we designed an oligonucleotide containing a single 8-oxoG. Using LC–MS intact mass, we identified a strand break directly 5′ and 3′ of the 8-oxoG that is specific to oxidized G under heat alkaline treatments (Supplementary text 1 and Supplementary Figure S3). Thus, the heat-alkaline treatment performed in RIMS-seq induced strand breaks at oxidative damage sites, preventing the amplification of 8-oxoG-containing fragments and de-facto decreasing the frequency of G to T in the RIMS-seq libraries.

A slight elevation of G to C and T to C read variants can be observed in RIMS-seq compared to DNA-seq but this damage is of low frequency and therefore is not expected to notably affect the sequencing performance QC of RIMS-seq.

We performed QC metrics and assemblies of Xp12 for all the alkaline-heat treatment conditions, including a control DNA-seq. The overall sequencing performances were assessed in terms of insert size, GC bias and genome coverage. Similar results were observed between RIMS-seq and the DNA-seq control at all treatment times, indicating that the RIMS-seq heat-alkaline treatment does not affect the quality of the libraries (Supplementary Figure S4).

We also evaluated the quality of the assemblies compared to the Xp12 reference genome and found that all conditions lead to a single contig corresponding to essentially the entire genome with very few mismatches (Supplementary Table S1). These results suggest that the heat-alkaline treatment does not affect the assembly quality, raising the possibility of using RIMS-seq for simultaneous *de novo* genome assembly and methylase specificity identification. We found that a 3-h treatment provides a good compromise between the deamination rate (resulting in ∼0.3% of m5C showing C to T transition) and duration of the experiment. We found that longer incubation times (up to 14 h) increased the deamination rate by up to 1% and decided this is a slight sensitivity increase compared to the additional experimental time required.

#### RIMS-seq is able to distinguish methylated versus unmethylated motifs in *E. coli*

To validate the application of RIMS-seq to bacterial genomes, we sequenced dcm+ (K12) and dcm- (BL21) *E. coli* strains. In K12, the DNA cytosine methyltransferase *dcm* methylates cytosine at CCWGG sites (C = m5C, W = A or T) and is responsible for all m5C methylation in this strain (19). *E. coli* BL21 has no known m5C methylation. Heat/alkaline treatments were performed at three time points (10 min, 1 h and 3 h). In addition, we performed a control experiment corresponding to the standard DNA-seq. Resulting libraries were paired-end sequenced using Illumina and mapped to their corresponding genomes (Methods).

**Figure 2.**
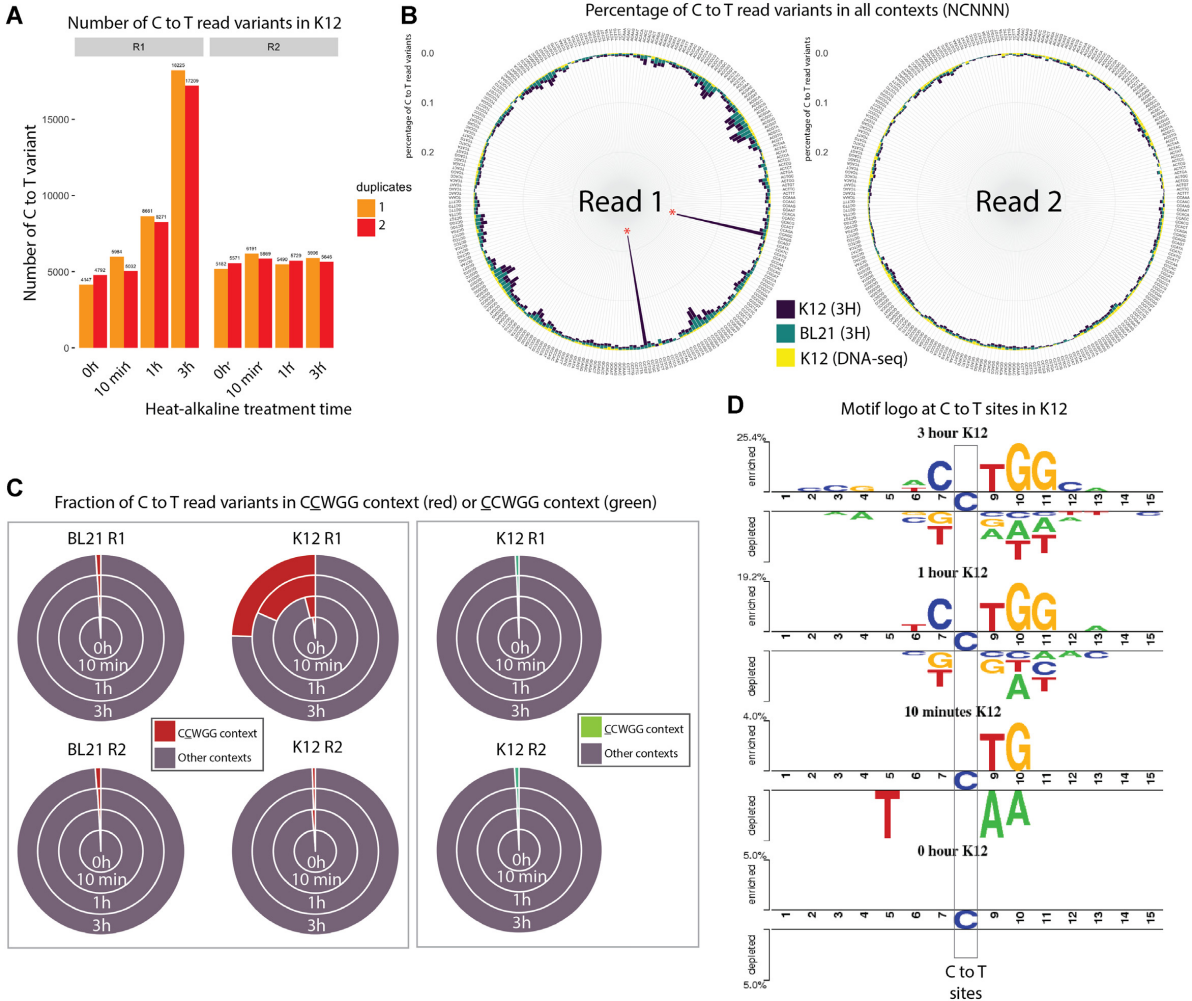
(**A**) Bar plots representing the number of C to T read variants for K12 in R1 and R2 after different heat/alkaline treatment times. Colors represent duplicate experiments. (**B**) Circular bar plots representing the percentage of C to T read variants in all NCNNN contexts (with N = A, T, C or G) for Read 1 (R1, left) and Read 2 (R2, right) in DNA-seq performed on K12 (yellow bars), RIMS-seq (3H) performed on BL21 (green) and RIMS-seq (3H) performed on K12 (dark blue). Red asterisks denote CCWGG contexts with W being either A or T. (**C**) Proportion of C to T read variants in CCWGG (red) or CCWGG (green) contexts compared to other NCNNN or CNNNN contexts for R1 and R2 in K12 and BL21. The C to T read variants in CCWGG and CCWGG motifs represent less than 2% of all variants except in K12 (R1 only) after 10 min, 1- and 3-h treatments where the CCWGG motifs represent 4.1%, 22.5% and 32.6% of all C to T read variants respectively. The increase of C to T read variants in the CCWGG context is therefore specific to R1 in K12 strain. (**D**) Visualization of the statistically significant differences in position-specific nucleotide compositions around C to T variants in R1 compared to R2 using Two Sample Logo (21) for the K12 sample subjected to (from top to bottom) 3 h, 1 h, 10 min and 0 min heat alkaline treatment.

For comparison, all datasets were downsampled to 5 million reads corresponding to 200× coverage of the *E. coli* genome and instances of high confidence C to T variants (*Q* score > 35) on either R1 or R2 were identified. As expected, control DNA-seq experiments show comparable numbers of C to T read variants between R1 and R2, indicating true C to T variants or errors during amplification and sequencing (Figure 2A). On the other hand, the overall number of C to T read variants in R1 is progressively elevated for 10 min, 1 h and 3 h of heat-alkaline treatment of the *E. coli* K12 samples with an overall 4-fold increase after 3 htreatment compared to no treatment; heat-alkaline treatments did not increase the rate of C to T read variants in R2 (Figure 2A). We anticipate that the elevation of the *E. coli* K12 C to T read variants in R1 is due to deamination of m5C. In this case, the elevation should be specifically found in Cs in the context of CCWGG (with the underlined C corresponding to the base under consideration). To demonstrate this, we calculated the fraction of C to T read variants in CCWGG compared to other contexts. We observed a large elevation of the C to T read variants in the CCAGG and CCTGG contexts for K12 (Figure 2B). As expected, the C to T read variants show no elevation at CCAGG and CCTGG contexts for the *E. coli* BL21 strain that is missing the *dcm* methylase gene (Figure 2B). Thus, this C to T read variant elevation is specific to the *E. coli* K12 strain subjected to heat-alkaline treatments, consistent with deamination detectable only on methylated sites. Taken together, these results indicate that the elevated rate of C to T variants observed in R1 from *E. coli* K12 is the result of m5C deamination in the CCWGG context.

Next, we assessed whether the difference in the C to T read variant context between R1 and R2 at the CCWGG motif provides a strong enough signal to be discernible over the background noise. For this, we calculated the fraction of C to T read variants in CCWGG and CCWGG compared to all the other NCNNN and CNNNN contexts, respectively. After 3 h of heat-alkaline treatment, the fraction of C to T read variants in a CCWGG context increased, rising from only 1.9% in regular DNA-seq to ∼25% of all the C to T variants. This increase is only observable in R1 of the K12 strain (Figure 2C). Conversely, no increase can be observed in a CCWGG context for which the C to T variant rate at the first C is assessed (Figure 2C). Thus, RIMS-seq identified the second C as the one bearing the methylation, consistent with the well described dcm methylation of *E. coli* K12 (20) (19), highlighting the ability of RIMS-seq to identify m5C methylation at base resolution within the methylated motif.

Next, we calculated significant (*P*-value < 0.01) differences in position-specific nucleotide compositions around C to T variants in R1 compared to R2 using Two Sample Logo (21). We found a signal consistent with the dcm methylase specificity in K12 RIMS-seq samples at 1 and 3 h of heat alkaline treatment (Figure 2D) demonstrating that it is possible to identify methylase specificities in genomic sequence subject to as little as 1 h of alkaline treatment. These results support the application of RIMS-seq for the *de novo* identification of methylase specificity at base resolution.

#### RIMS-seq identifies the correct methylase specificity *de novo* in *E. coli* K12

**Figure 3.**
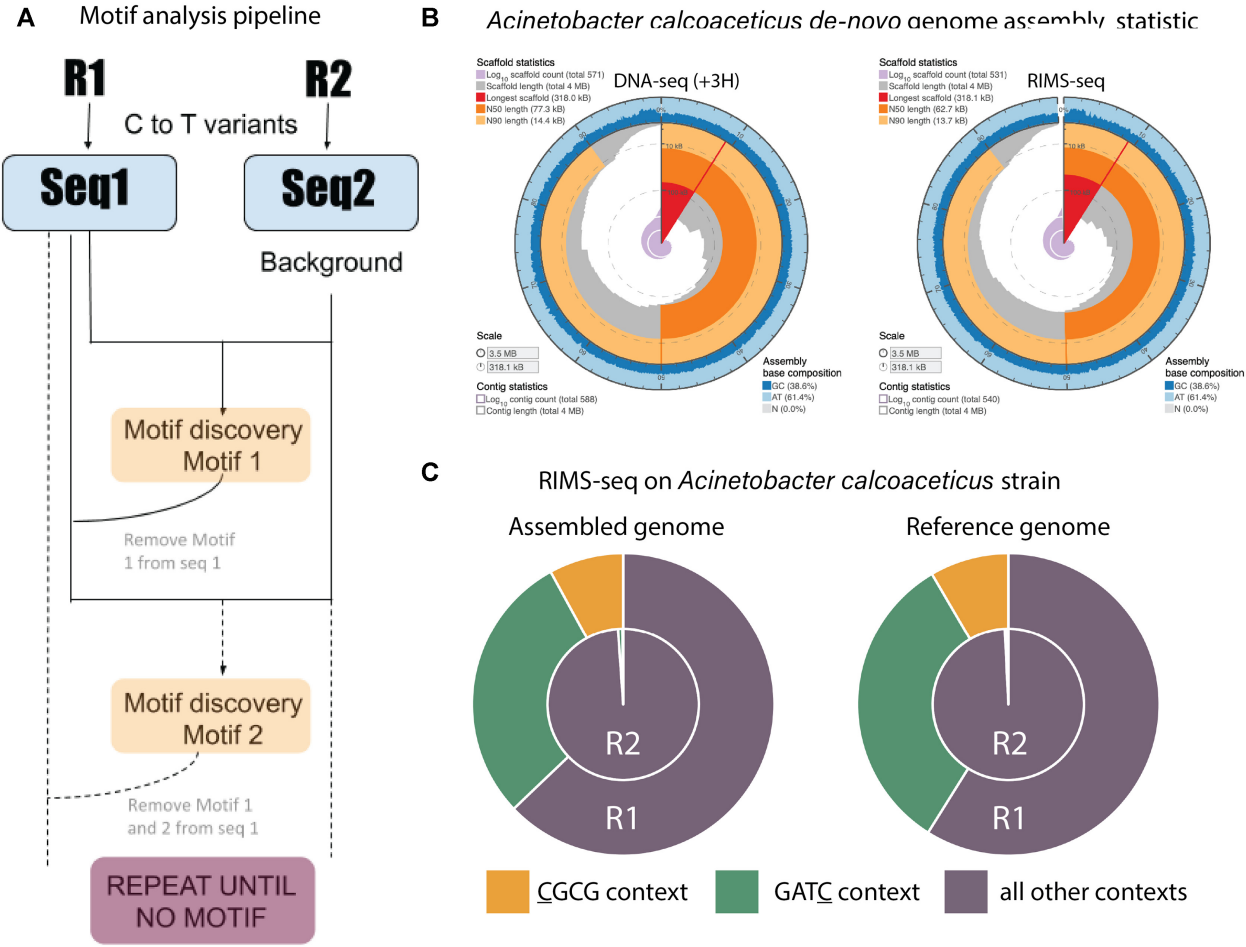
*De novo* discovery of methylase specificity using RIMS-seq. (**A**) Description of the RIMS-seq motif analysis pipeline. First, C to T read variants are identified in both Read 1 and Read 2 separately. Then, the MosDI program searches for overrepresented motifs. Once a motif is found, the pipeline is repeated until no more motifs are found, enabling identification of multiple methylase specificities in an organism. (**B**) Assembly statistics obtained using the sequence from the standard DNA-seq (+3H, left) and RIMS-seq (right). Visualization using assembly-stats program (https://github.com/rjchallis/assembly-stats). The corresponding table with the statistical values is available in the supplementary material (Supplementary Table S2). (**C**) Fractions of C to T read variants in CGCG (yellow) or GATC (green) contexts compared to other contexts for R1 and R2 in Acinetobacter calcoaceticus ATCC 49823 using the assembled or the reference genome. The increase of C to T read variants in these contexts are similar when using either the assembled or reference genomes

In order for RIMS-seq to identify methylase specificities *de novo*, we devised an analysis pipeline based on MoSDi (22) to find sequence motif(s) that are over-represented around C to T transitions in R1 reads (Figure 3A, analysis pipeline available at https://github.com/Ettwiller/RIMS-seq). In brief, the pipeline extracts the sequence context at each C to T read variant in R1 (foreground) and R2 (background). MoSDi identifies the highest over-represented motif in the foreground sequences compared to the background sequences. To accommodate the presence of multiple methylases in the same host, the first motif is subsequently masked in both the foreground and background sequences and the pipeline is run again to find the second highest over-represented motif and so on until no significant motifs can be found (see Materials and Methods for details). Running the pipeline using the K12 strain RIMS-seq data identifies one significant over-represented motif corresponding to the CCWGG motif (*P*-value = 9.71e^−77^, 4.25e^−858^ and 3.61e^−4371^ for 10, 60 and 180 min of alkaline treatment respectively) with the cytosine at position 2 being m5C.

Summing up, we devised a novel sequencing strategy called RIMS-seq and its analysis pipeline to identify m5C methylase specificity *de novo*. When applied to *E. coli* K12, RIMS-seq identifies the dcm methylase specificity as CCWGG with the methylated site located on the second C, consistent with the reported dcm methylase specificity (Table 1).

#### RIMS-seq identifies multiple methylase specificities *de novo* within a single microorganism

To assess whether RIMS-seq can identify methylase specificity in strains expressing multiple methylases, we repeated the same procedure on a strain of *Acinetobacter calcoaceticus* ATCC 49823 expressing two m5C methylases with known specificities (4). RIMS-seq identifies CGCG (*P*-value = 2.33e^–^^174^) and GATC (*P*-value = 3.02e^–^^1308^) (Table 1) both motifs have been confirmed by MFRE-seq (10). Thus, RIMS-seq is able to *de novo* identify methylase specificities in bacteria expressing multiple methylases.

#### RIMS-seq can be applied for genome sequencing and m5C profiling in bacteria without a reference genome

We investigated whether RIMS-seq can be used to identify methylase specificities of uncharacterized bacteria for which a reference genome is unavailable. More specifically, we evaluated if the reads generated using RIMS-seq can be used for both identifying methylase specificities and generating an assembly of comparable quality to DNA-seq.

For this, we performed RIMS-seq on *A. calcoaceticus* ATCC 49823 genomic DNA as described above as well as a control DNA-seq experiment for which the alkaline treatment was replaced by 3 h incubation in TE (DNA-seq(+3H)). We compared the *de novo* assembly obtained from the reads generated by the DNA-seq(+3H) and the *de novo* assembly obtained from the reads generated by RIMS-seq (see Materials and Methods). In brief, the alkaline treatment did not alter the important metrics for assembly quality such as the rate of mismatches and N50 demonstrating that the elevated C to T variant rate at methylated sites is not high enough to cause assembly errors (Figure 3B).

We then proceeded to map the RIMS-seq reads to the assembly and motifs were identified using the RIMS-seq *de novo* motif discovery pipeline. As expected, the same motifs found when mapping to the reference genome are also found in the *A. calcoaceticus de novo* assembly with similar significance (GATC (*P*-value = 1.44e^−1255^) and CGCG (*P*-value = 8.6e^−228^) (Figure 3C). These motifs correspond to the methylase specificities expected in this strain indicating that RIMS-seq can be applied for genome sequencing and assembly of any bacterium without the need for a reference genome.

#### RIMS-seq can be complemented with SMRT sequencing to obtain a comprehensive overview of methylase specificities

RIMS-seq performed in parallel with SMRT sequencing has the advantage of comprehensively identifying all methylase specificities (m5C, m4C and m6A methylations) and results in an assembly of higher quality than with short reads illumina data. We applied this hybrid approach to *Acinetobacter calcoaceticus* ATCC 49823 for which a SMRT sequencing and assembly had been done previously (4). RIMS-seq was performed as described above and the reads were mapped to the genome assembly obtained from SMRT-sequencing. We again found the two m5C motifs: CGCG (*P*-value = 1.84e^−1535^) and GATC (*P*-value = 4.93e^−6856^) from the RIMS-seq data in addition to the 13 m6A motifs described previously using SMRT sequencing (4). This result demonstrates the advantage of such a hybrid approach in obtaining closed genomes with comprehensive epigenetic information.

#### XP12 can be used as a spiked-in to measure the deamination rate

To ensure the correct level of heat-alkaline deamination rate, XP12 can be used as spiked-in to measure the deamination rate at m5C. To illustrate the practicality of such control, we subjected Haemophilus influenzae Rd ATCC 51907 (Table 1) spiked-in with XP12 DNA to various NaOH concentrations and treatment times. We observed deamination rates varying from 0.24% (0.1 M NaOH, 3 h) to 2.72% (0.5 M NaOH, 3 h) (Supplementary Figure S2C). We further investigated the error rates in both the bacteria and XP12 for substitutions other than C to T at various heat alkaline conditions (Supplementary Figure S2C) and found that all substitution rates are comparable to the rates obtained using standard DNA-seq. Taken together, these results indicate that the heat alkaline treatments in the measured ranges are not expected to notably affect the sequencing performance QC in bacteria.

### RIMS-seq can be applied to a variety of RM systems

Methylases targets are usually palindromic sequences between 4 nt and 8 nt, and a single bacterium often possesses several, distinct MTase activities (23). Next, we tested the general applicability of RIMS-seq and the *de novo* motif discovery pipeline using bacterial genomic DNA from our in-house collections of strains.

For some bacterial strains, the methylase recognition specificities have been previously experimentally characterized. In all of those strains, RIMS-seq confirms the specificities and identifies the methylated cytosine at base resolution (Table 1). We have tested the identification of 4-mers motifs such as GATC, CGCG (*Acinetobacter calcoaceticus*) and GCGC (*Haemophilus parahaemolyticus*) up to 8-mers motifs such as ACCGCACT and AGTGCGGT (*Haemophilus influenzae*). Motifs can be palindromic or non-palindromic (Table 1 and Supplementary Table S3). In the latter case, RIMS-seq defines non-palindromic motifs at strand resolution. For example, RIMS-seq identifies methylation at two non-palindromic motifs ACCTGC as well as its reverse complement GCAGGT in the *Bacillus fusiformis* strain (Table 1).

**Figure 4.**
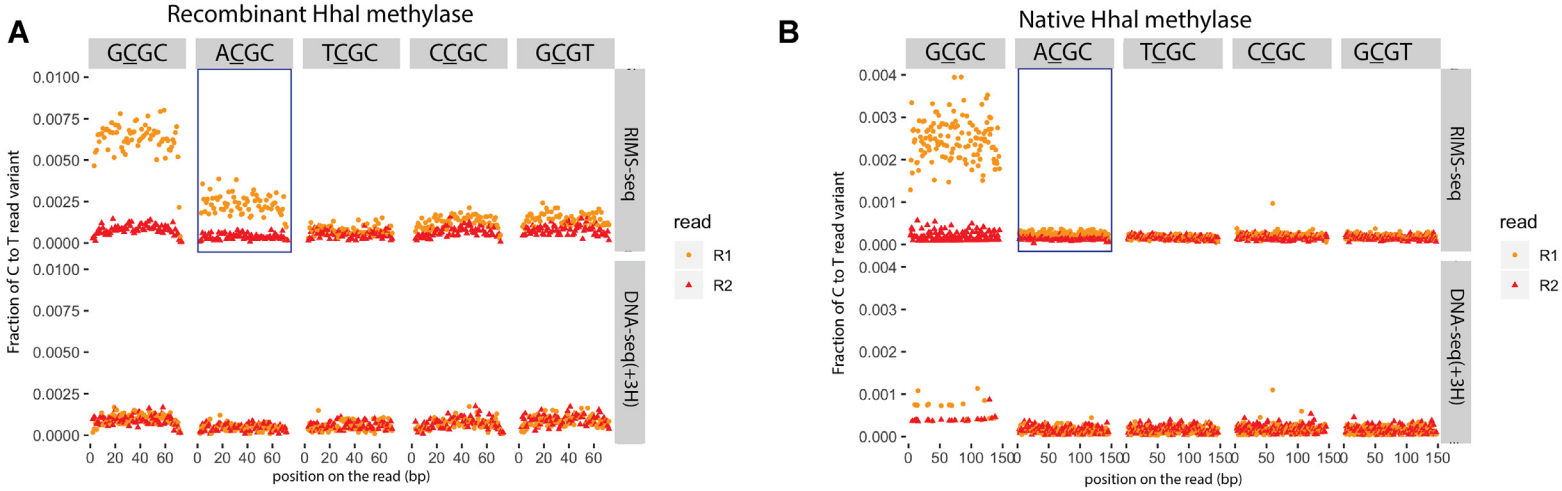
C to T error profile in GCGC (canonical recognition site), ACGC, TCGC, CCGC and GCGT. in R1 reads (orange) and R2 reads (red) for RIMS-seq (upper panel) and DNA-seq(+3H) (lower panel) *A*. Recombinant HhaI methylase expressed in *E. coli B*. Native HhaI methylase expressed in *Haemophilus parahaemolyticus*. Elevation of C to T in the R1 read variant can be observed in the context of GCGC for both the recombinant and native HhaI genomic DNA and in the context of ACGC only for DNA from the recombinant but not the native HhaI.

A number of RM systems have been expressed in other hosts such as *E. coli* for biotechnological applications. For the methylase M.HhaI recognizing GCGC (4), we performed RIMS-seq and a control DNA-seq(+3H) on both the native strain (*Haemophilus parahaemolyticus* ATCC 10014) and in *E. coli* K12 expressing the recombinant version of M.HhaI. Interestingly, we found that the *de novo* RIMS-seq analysis algorithm identifies RCGC (with R being either A or G) for the recombinant strain and GCGC for the native strain (Figure 4A). Conversely, no notable elevation of C to T read variants are observed at ACGC for the native strain (Figure 4B), confirming the *de novo* motif discovery results from the analysis pipeline. Collectively, these results suggest that the recombinant methylase shows star activity, notably in the context of ACGC, that is not found in the native strain. We hypothesize that the star activity is the result of the over-expression of the methylase in *E. coli* K12. Interestingly, ACGC is not a palindrome motif and consequently the star activity results in hemi-methylation of the ACGC sites and not the GCGT motif.

### RIMS-seq can be applied to microbial communities

We assessed whether RIMS-seq can be applied to mixed microbial communities using synthetic gut and skin microbiomes from ATCC containing 12 and 6 bacterial species, respectively. We also complemented the RIMS-seq experiment with the control experiment DNA-seq(+3H) and a bisulfite treatment to validate the RIMS-seq findings. Reads were mapped to their respective microbiome reference genomes (Materials and Methods). For the gut microbiome we found a mapping rate (properly paired only) of 95.79%, 95.77% and 66.2% for RIMS-seq, DNA-seq and bisulfite-seq respectively. Concerning the skin microbiome, 85.89%, 85.35% and 54.9% of reads were mapped for RIMS-seq, DNA-seq and bisulfite-seq respectively. The low mapping rate for bisulfite-seq is a known challenge as the reduction of the alphabet to A, G, T generates ambiguous mapping (24).

**Figure 5.**
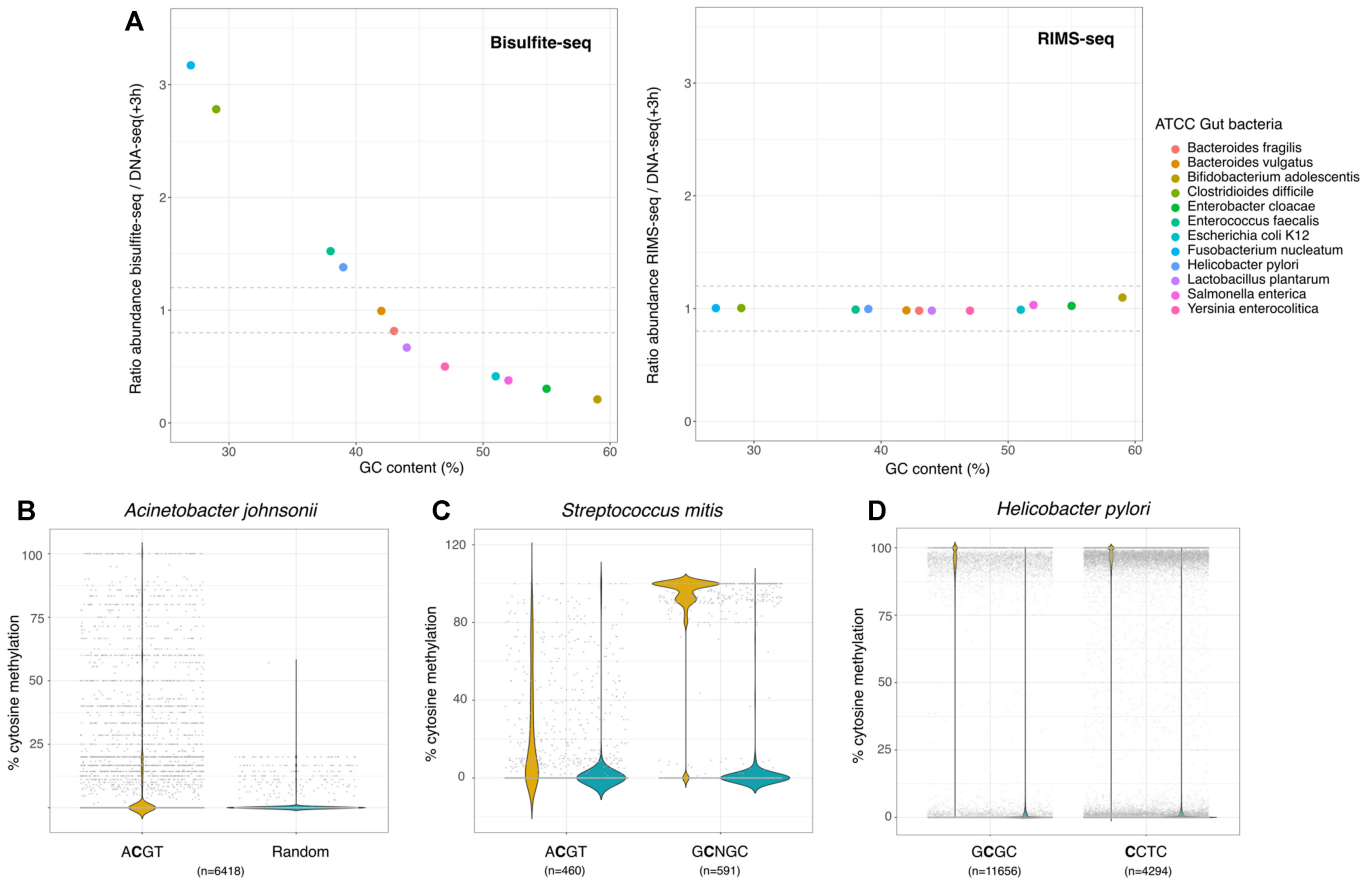
(**A**) Bacterial abundance in the ATCC gut microbiome calculated from bisulfite-seq data (left) and RIMS-seq (Right) normalized to DNAseq(+3H). The normalized abundance is plotted relative to the GC content of each bacterium. (**B**) Methylation levels in *Acinetobacter johnsonii* (ATCC skin microbiome).The methylation level was calculated for cytosine positions in the context of ACGT (yellow) and randomly selected positions in other contexts (blue). These bisulfite-seq data suggest some sites are methylated in the context of ACGT, but they are not fully methylated. (**C**) Methylation level in Streptococcus mitis (ATCC skin microbiome) calculated from bisulfite-seq data. The methylation level was calculated for cytosine positions in the context of ACGT and GCNGC (yellow) as well as for randomly selected positions in other contexts (blue). (**D**) Methylation level in Helicobacter pylori (ATCC gut microbiome) calculated from bisulfite-seq data. The methylation level was calculated for cytosine positions in the context of GCGC and CCTC (yellow) as well as for randomly selected positions in other contexts (blue).

To use RIMS-seq as an equivalent to DNA-seq for mixed community applications, RIMS-seq should produce sequencing quality metrics that are similar to standard DNA-seq, especially on the estimation of species relative abundances. We therefore compared RIMS-seq sequencing performances with DNA-seq(+3H) and bisulfite sequencing. We found that bisulfite sequencing elevates abundances of AT-rich species such as *Clostridioides difficile* (71% AT), *Enterococcus faecalis* (63% AT) and *Fusobacterium nucleatum* (73% AT) (Figure 5A, Supplementary Figure S5). For example, bisulfite sequencing over-estimated the presence of *Clostridioides difficile* by a factor of 2.65 and *Staphylococcus epidermidis* by a factor of 3.9 relative to DNA-seq. This over-estimation of an AT rich genome by bisulfite is a known bias of bisulfite sequencing and relates to damage at cytosine bases (25). Conversely, we found that the species abundances are similar between DNA-seq(+3H) and RIMS-seq (abundance ratios between 0.8 and 1.2) indicating that RIMS-seq can be used to quantitatively estimate microbial composition.

#### RIMS-seq identifies known and novel methylase specificities in synthetic microbial communities

Overall, we found motifs for 6 out of the 12 gut microbiome species and five out of the six skin microbiome species (Supplementary Table S3). The motifs range from four to eight nucleotides long and 70% are palindromic. Interestingly, we found an unknown palindromic motif GGCSGCC (with S being either C or G) from *Micrococcus luteus* (NC_012803.1) in the skin community. To our knowledge, this is the first time this 7nt motif is identified, showing the potential of RIMS-seq to identify new methylase specificities. Results obtained with RIMS-seq were also validated using bisulfite sequencing. RIMS-seq identified two motifs in *Helicobacter pylori* from the ATCC synthetic gut microbiome: GCGC as well as an additional non-palindromic motif CCTC that was identified by the bisulfite analysis pipeline as CYTC with Y being either C or T. The CCTC motif is very common in *Helicobacter pylori*s species, it has been described to be modified at m5C on one strand, while modified at m6A on the other strand (4). In order to confirm the RIMS-seq motif, we investigated the bisulfite-seq data and compared the methylation level in cytosines present in the CCTC context versus cytosines in any other context. We see a methylation level above 90% at the cytosines in the CCTC context confirming the existence of this methylated motif in *Helicobacter pylori* (Figure 5D). Interestingly, m4C methylation in *Helicobacter pylori* has been shown to also occur at TCTTC (26), resulting in the composite motif CYTC (TCTTC and NCCTC) found in the bisulfite data. Contrary to bisulfite, RIMS-seq does not identify m4C methylation (27), hence the identification of the CCTC motif instead.

Also, interestingly, bisulfite-seq results indicate that the ACGT motif in *Acinetobacter johnsonii* and *Streptococcus mitis* from the ATCC synthetic skin microbiome are not fully methylated (Figure 5B). Most of the sites in *Acinetobacter johnsonii* show a methylation of about 10% while in *Streptococcus mitis*, the average methylation per site is 23% (Figure 5C). These results highlight that despite the low methylation levels, RIMS-seq is able to detect the ACGT motif at high significance (*P*-value < 1e^−100^). We took advantage of the fact that Streptococcus mitis has two methylated motifs, ACGT and GCNGC with an average methylation per site at 23% and 91% respectively (Figure 5C) to evaluate the sequencing depth required for RIMS-seq to *de-novo* identify both motifs. As expected, the fully methylated GCNGC motif is found using 4 times fewer sequencing reads than the ACGT motif, with a required 1 million and 4 million mapped reads respectively (Supplementary Figure S6A and B).

## DISCUSSION

In this study, we developed RIMS-seq, a sequencing method to simultaneously obtain high quality genomic sequences and discover m5C methylase specificity(ies) in bacteria using a single library preparation. The simplicity of the procedure makes RIMS-seq a cost effective and time saving method with only an additional 3 h sodium hydroxide incubation and an additional column-based cleaning step. Theoretically, the cleaning step can be avoided if a small volume of the library is used for the amplification step, but we have not tested this procedure. By increasing the sodium hydroxide concentration to 0.5M or even 1M, the incubation time can be reduced to 30 min.

Due to the limited deamination rate, RIMS-seq is equivalent to short read DNA-seq in terms of sequencing quality. Sequencing QC metrics such as coverage, GC content and mapping rate are similar for RIMS-seq and DNA-seq. Thus, RIMS-seq can be used for applications such as, but not limited to, shotgun sequencing, genome assembly and estimation of species composition of complex microbial communities. This dual aspect of RIMS-seq is analogous to SMRT sequencing for which methylation is inferred from the IPD ratio. We showed that both PacBio and RIMS-seq can be complementary with the ability to obtain a complete methylome: m6A and m4C methylase specificities can be obtained from SMRT sequencing while m5C methylase specificity can be obtained from RIMS-seq. Combining both sequencing technologies also allows for a hybrid assembly strategy resulting in closed reference genomes of high sequencing accuracy.

We applied RIMS-seq to several bacteria and identified a variety of methylation motifs, ranging from 4 nt to 8 nt long, palindromic and non-palindromic. Some of these motifs were identified for the first time, demonstrating the potential of the technology to discover new methylase specificities, from known as well as from unknown genomes. We also validated that RIMS-seq can identify multiple methylase specificities from a synthetic microbial community and estimate species abundances. However, RIMS-seq has caveats similar to metagenomics sequencing when applied to study natural microbial communities. Closely related species are likely to co-exist and assigning the motif to the correct species can be challenging. Furthermore, single nucleotide polymorphisms found in microbial communities may confound the identification of the C to T deamination, increasing the background noise for the detection of motifs. Finally, species in microbiomes are unevenly represented which can cause RIMS-seq to identify motifs only in the most abundant species.

Because RIMS-seq is based on a limited deamination, it requires the combined signal over many reads to be large enough to effectively identify methylase specificity. For the vast majority of the methylases in RM systems, methylation is present at enough sites across the genome for RIMS-seq to determine their specificities. Nonetheless, bacterial methylases can be involved in other processes such as, but not limited to, DNA mismatch repair (28), gene regulation (29) and sporulation (30) and the recognition sites may not necessarily be fully methylated. Partially methylated sites can be found using RIMS-seq but more analysis needs to be done to evaluate how pervasive methylation needs to be to provide a RIMS-seq signal. In other cases, methylated motifs are too specific or under purifying selection, resulting in just a handful of sites in the genome. In these cases, RIMS-seq signals can only be obtained with enough read coverage to compensate for the scarcity of those sites. While the methylase specificities are of great interest in bacteria due to their diversity in recognition sequences, applying RIMS-seq to humans would lead to the identification of the already well-described CpG context. In this case, other technologies such as EM-seq or bisulfite-seq are more appropriate as they enable the precise genomic location to be obtained.

In summary, RIMS-seq is a new technology allowing the simultaneous investigation of both the genomic sequence and the methylation in prokaryotes. Because this technique is easy to implement and shows similar sequencing metrics to DNA-seq, RIMS-seq has the potential to substitute DNA-seq for microbial studies.

## DATA AVAILABILITY

The data have been deposited with links to BioProject accession number PRJNA706563 in the NCBI BioProject database (https://www.ncbi.nlm.nih.gov/bioproject/).

Custom-built bioinformatics pipelines to analyse sequencing reads from RIMS-seq are available at https://github.com/Ettwiller/RIMS-seq/.

## Supplementary Material

gkab705_Supplemental_File
