# Supplementary material for: Rapid identification of methylase specificity (RIMS-seq) jointly identifies methylated motifs and generates shotgun sequencing of bacterial genomes
Source: Nucleic Acids Res. 2021 Aug 20;49(19):e113. doi: 10.1093/nar/gkab705 (PMC8565308; doi:10.1093/nar/gkab705)
Supplement: gkab705_Supplemental_File [file gkab705_supplemental_file.pdf]

Supplementary material : Rapid Identification of Methylase Specificity (RIMS-seq) jointly identifies methylated motifs and generates shotgun sequencing of bacterial genomes

Chloé Baum, Yu-Cheng-Lin, Alexey Fomenkov, Brian P. Anton, Lixin Chen, Bo Yan, Thomas C. Evans Jr, Richard J Roberts, Andrew C Tolonen, Laurence Ettwiller

Supplementary text 1 :

We designed an oligonucleotide containing a single 8-oxo-dG (/5FAM/TGGAGATTTTGATCACGGTAACC/i8oxodG/ATCAGAATGACAACAAGCCCGAATTCACCCAGGAGG/3Rox_N/). 50 uM of this 8-oxo-dG containing oligonucleotide was treated with 0.1M NaOH at 60C for 3 hours or 16 hours, respectively. Following alkaline and heat treatment, all reactions were neutralized with acetic acid and a clean-up with Monarch PCR & DNA cleanup kit (NEB, Ipswich). All cleanup DNA eluted with 20 ul of water were subjected for LC-MS analysis. Untreated 8-oxo-dG oligonucleotide was also subjected to LC-MS in the same run (**material and Methods**).

In the two alkaline and heat treatment conditions tested, LC-MS confirms two DNA fragments which are the strand-breaks products at 8-oxo-dG: 1) 7692.309 Dalton, which is the mass of 5'FAM-DNA fragment with a 3'-phosphate end before 8-oxo-dG; 2) 11867.1510 Dalton, which are the mass of 3' ROX-DNA fragment with a 5'-phosphate end after 8-oxo-dG. A 11139.888 Dalton peak was also detected, which is the 3'-DNA fragment without 3'Rox. Conversely, the untreated 8-oxo-dG oligonucleotide LC-MS shows a single peak corresponding to the intact oligonucleotide (**Supplementary Figure 3**).

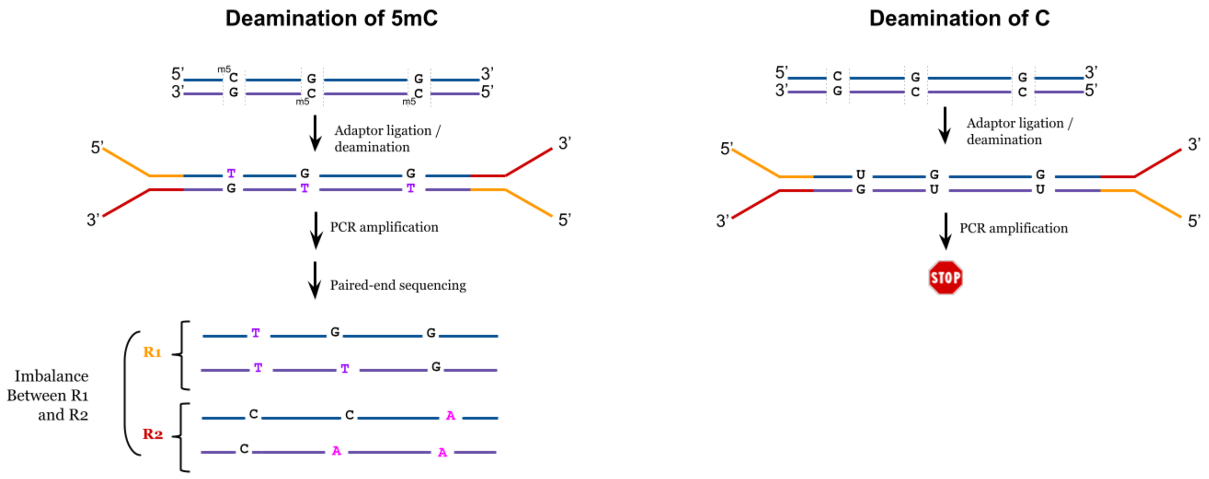

Supplementary Figure 1 : Schema describing the sequencing of 5mC and C containing DNA fragments. After DNA fragmentation and adaptor ligation (see methods), DNA fragments are subjected to a limited deamination under heat alkaline conditions. Deamination converts m5C to T (left schema) and C to U (right schema). U is a blocking damage for the polymerase, thus fragments containing U will not be amplified and sequenced. Conversely, fragments containing

deaminated 5mC (T) are amplified and sequenced leading to C to T variants. Because of the sequencing directionality of Illumina library, sequencing from the forward adaptor (Read 1) corresponds to the original strand; while sequencing from the reverse adaptor (Read2), corresponds to the reverse complement of the original strand. Thus, Read 1 shows an excess of C to T read variants compared to Read 2 (that shows an excess of G to A variant instead) leading to an imbalance of C to T/G to A variants. This imbalance is directly proportional to the deamination rate. In fragments containing a mixture of C and 5mC, the C to T/G to A imbalance can only be observed at methylated sites.

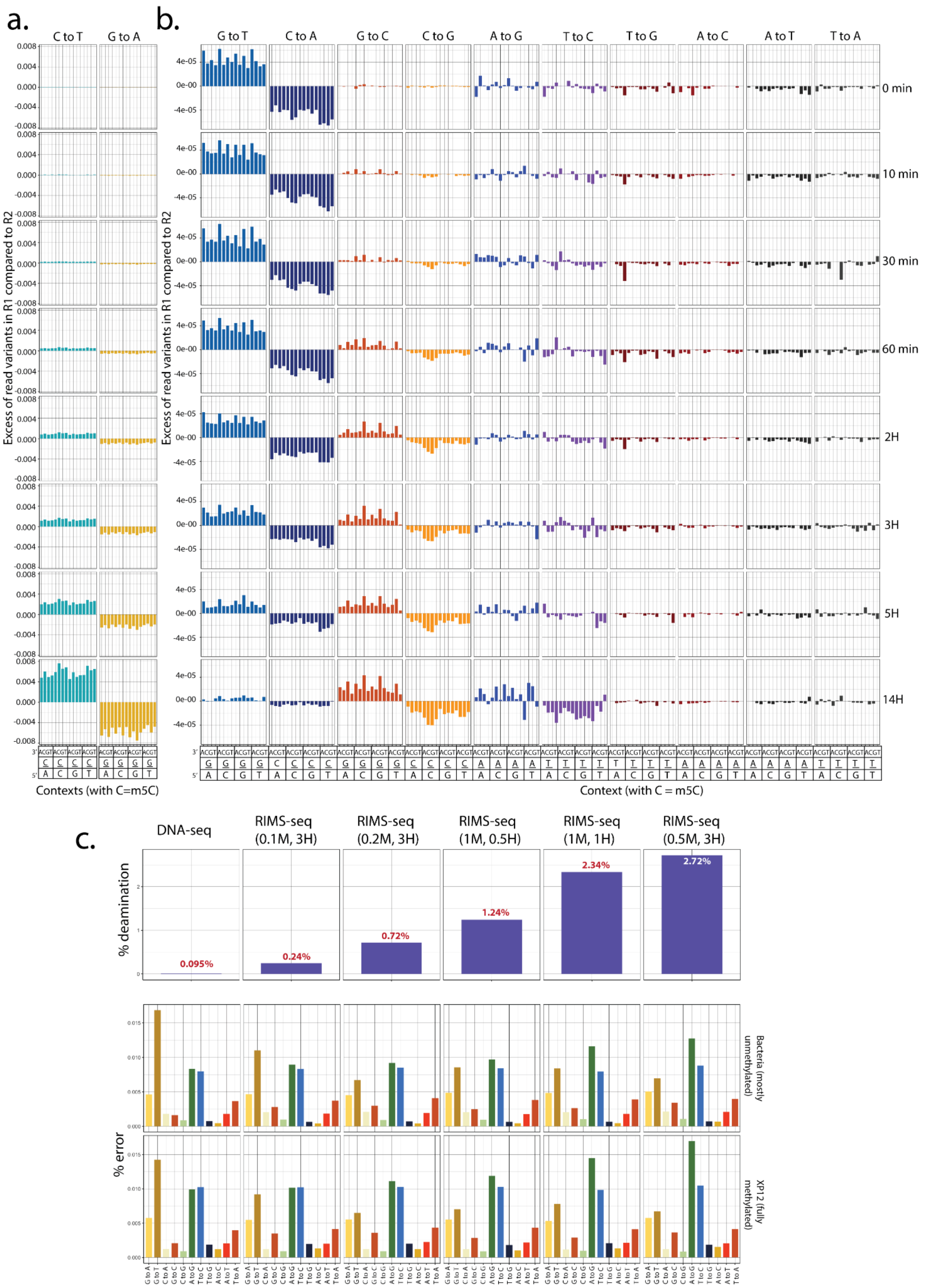
a.
C to T
G to A
Excess of read variants in R1 compared to R2
Contexts (with C=m5C)
b.
G to T
C to A
G to C
C to G
A to G
T to C
T to G
A to C
A to T
T to A
0 min
10 min
30 min
60 min
2H
3H
5H
14H
Excess of read variants in R1 compared to R2
Context (with C = m5C)
c.
DNA-seq
RIMS-seq (0.1M, 3H)
RIMS-seq (0.2M, 3H)
RIMS-seq (1M, 0.5H)
RIMS-seq (1M, 1H)
RIMS-seq (0.5M, 3H)
% deamination
0.095%
0.24%
0.72%
1.24%
2.34%
2.72%
% error
Bacteria (mostly unmethylated)
XP12 (fully methylated)

Supplementary Figure 2 Imbalance indicative of damage between R1 and R2 in a fully methylated genome (XP12) after RIMS-seq. **a.** Excess of read variants in R1 compare to R2 for control (t=0) and various heat alkaline treatment times (t= 10, 30 minutes, 1,2,3,5 and 14 hours) for C to T and G to A. G to A values are mirroring C to T because of the imbalance. **b.** Same as **a.** for all the other substitutions. Note that the Y-axis scale between **a.** and **b.** is different and the C to T excess in R1 is up to a 100 fold greater than for the other substitutions. Time scale represents the heat-alkaline incubation time (in minutes or hours). X-axis represents the different genomic context NNN context with N being A, T, m5C or G. c. Deamination rates (calculated as the % of C to T in excess in R1 compared to R2 in XP12) for 0.1, 0.2, 0.5 and 1M NaOH 60 degree C at various times (ranging from 0.5 to 3 hours, top panels) and the respective error rate for each substitution (calculated as the % of read variants, bottom panels) for bacteria (Haemophilus influenzae Rd ATCC 51907, mostly unmethylated) and XP12 (fully methylated).

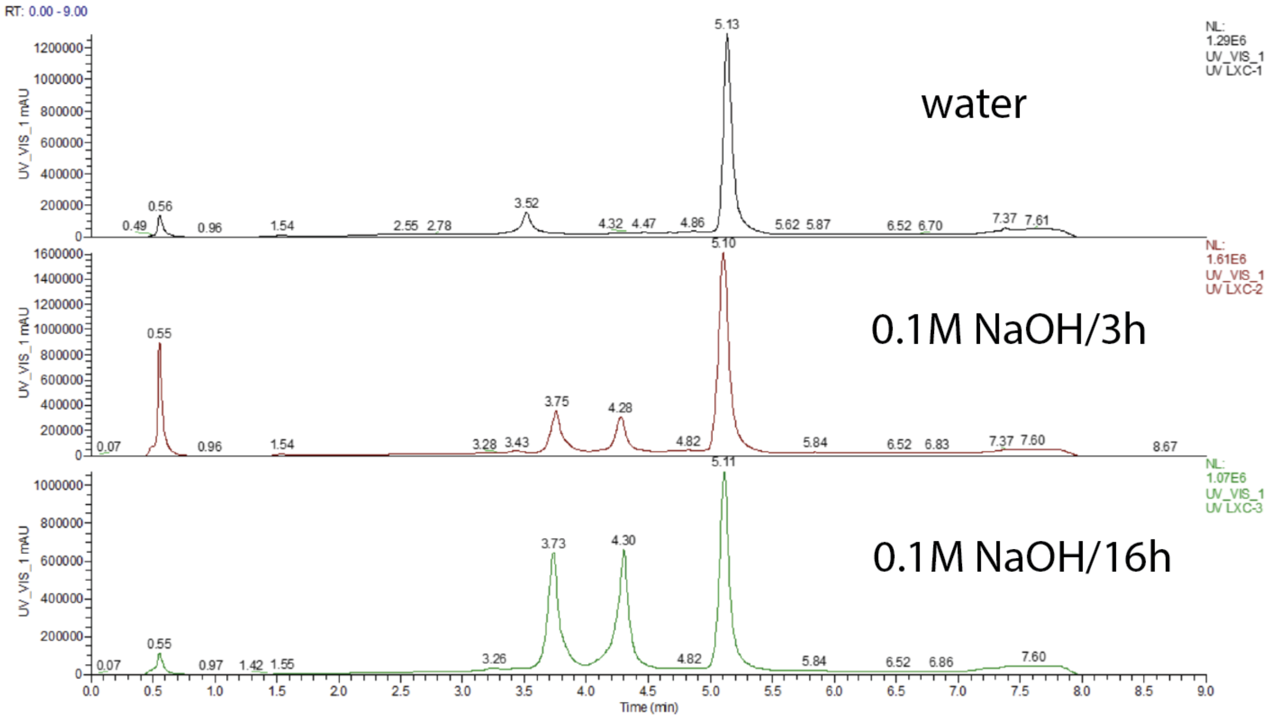

Supplementary Figure 3 : LC-MS result

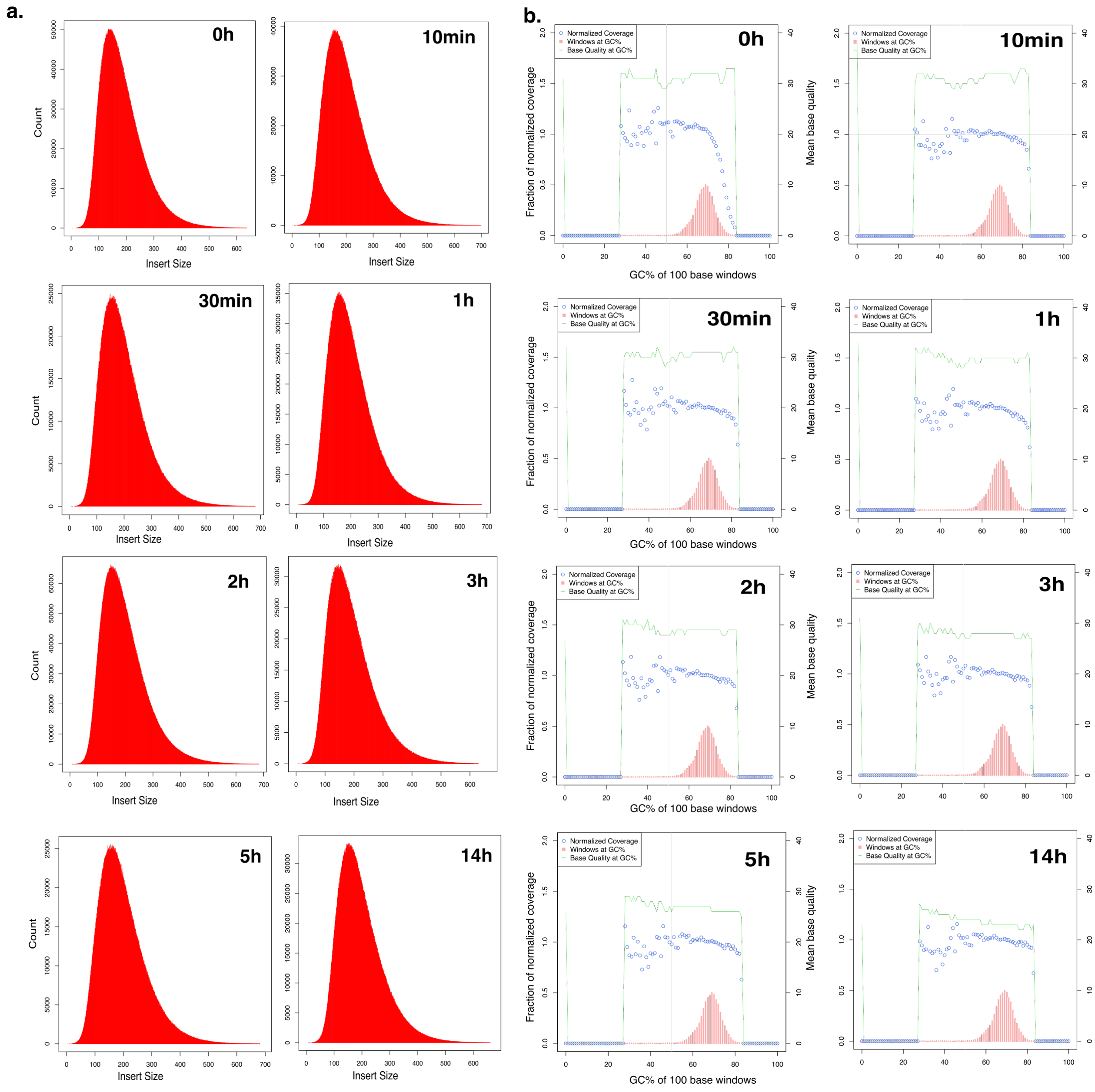

Supplementary Figure 4: Quality control of the sequencing performances for Xp12 gDNA

a. Insert size distribution (bp) for the control (0h) and different heat-alkaline treatment times. Input genomic DNA was sheared by Covaris treatment (200 bp target) and no further size selection was applied.

b. GC bias for the control (0h) and different heat-alkaline treatment times.

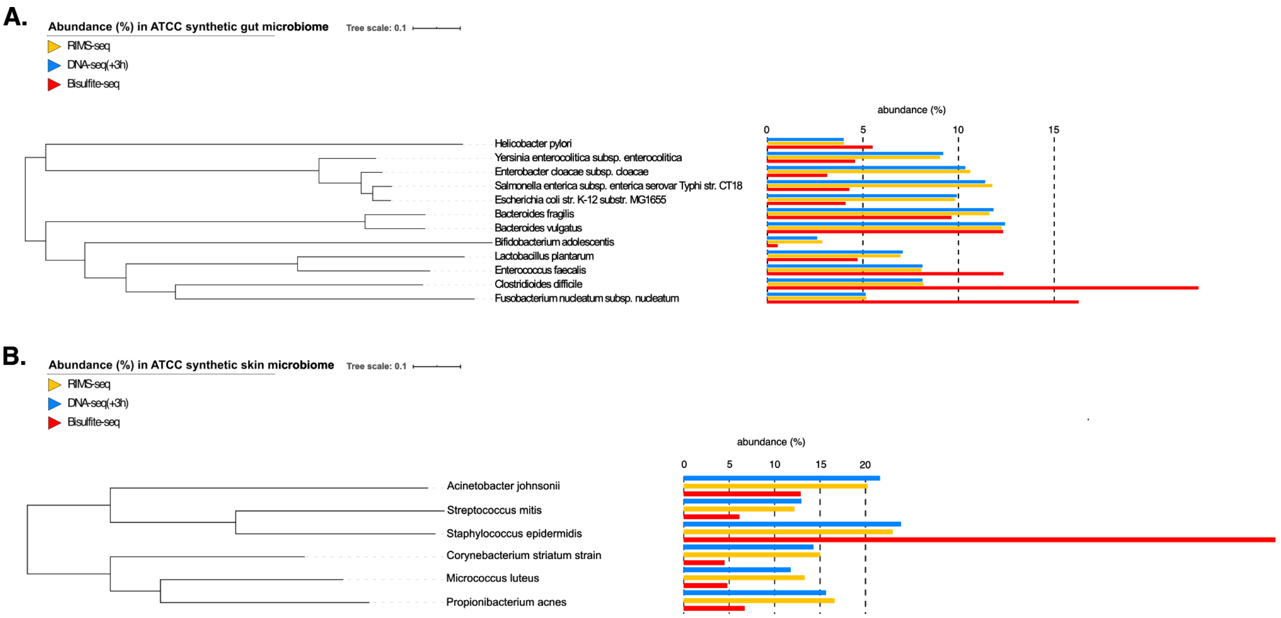

Supplementary Figure 5: Phylogenetic tree and barplot showing the relative abundance of each species in the ATCC synthetic (A.) gut and (B.) skin microbiome for RIMS-seq (yellow), DNA-seq(+3H, blue) and bisulfite sequencing (red). The relative abundance is calculated using the number of reads mapping to each species normalized to the total number of mapped reads.

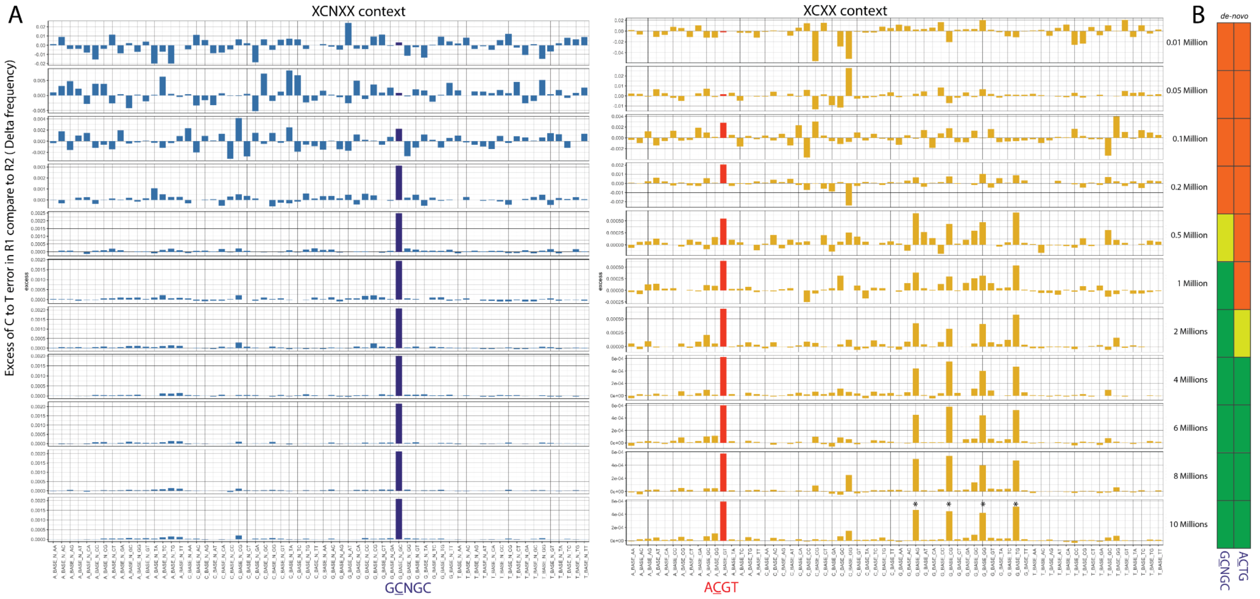

Supplementary Figure 6: A. Barplots showing the excess of C to T error in R1 compared to R2 (y-axis) in XCXX (left) and XCNXX (right) contexts (with X=either A,T,C or G and N being any nucleotides). The dataset has been downsampled to various numbers of read mapping to Streptococcus mitis ranging from 0.01 (top) to 10 millions (bottom) reads. The ACGT and GCNGC context is highlighted in red and dark blue respectively. Asterix (*) denote the GCAGN,

GCTGN,GCCGN,GCGGN motifs which correspond to a subset of the GCNGC motif. **B.** De-novo identification of the ACGT and GCNGC motifs. Red denotes motif not found, yellow denotes the correct motif was found with p-values between 1e-50 and 1e-100 and green denotes the correct motif was found with p.values < 1e-100.

Alakline-heat treatment time	0h	10min	30min	1h	2h	3h	5h	14h
**Statistics without reference genome**								
nb contigs	1	1	1	1	1	1	1	1
largest contig	63783	63882	64181	63774	63873	63839	63873	63774
**Statistics with reference genome**								
largest alignment	63783	63782	63774	63774	63285	63839	63773	63774
total aligned length	63783	63782	63774	63774	63285	63839	63773	63774
GC %	68.17	68.17	68.17	68.17	68.17	68.18	68.17	68.17
N50	63783	63882	63774	63774	63873	63839	63873	63774
Genome fraction (%)	99.239	99.238	99.225	99.225	99.224	99.241	99.224	99.225
% reads mapping back to assembly	99.75	99.73	99.8	99.76	99.82	99.78	99.75	99.84
**Misassemblies**								
nb misassemblies	0	0	0	0	0	0	0	0
misassembled contig length	0	0	0	0	0	0	0	0
local misassemblies	1	1	1	1	1	1	1	1
**Mismatches**								
N's per 100kbp	0	156.14	0	0	156.56	0	156.56	0
nb mismatches per 100kbp	3.14	3.14	1.57	1.57	3.14	4.7	1.57	3.14
nb indels per 100kbp	0	0	0	0	0	0	0	0

Supplementary Table 1: Xp12 assembly statistics for various heat/alkaline treatment times (see material and methods)

	dnaseq 3h	RIMS	reference
span (bp)	3,540,609	3,515,857	3,543,981
N (%)	0.02	0.01	0.00
GC (%)	38.62	38.59	38.31
AT (%)	61.38	61.41	61.69
scaffold count	571	531	1
longest scaffold (bp)	317,976	318,149	3,543,981
scaffold N50 length (bp)	77,278	62,717	3,543,981
scaffold N50 count	14	15	1
scaffold N90 length (bp)	14,355	13,679	3,543,981
scaffold N90 count	52	59	1
contig count	588	540	1
contig N50 length (bp)	62,601	58,046	3,543,981
contig N50 count	15	16	1
contig N90 length (bp)	13,997	13,455	3,543,981
contig N90 count	57	63	1

Supplementary Table 2 : Assembly statistics for *Acinetobacter calcoaceticus* ATCC 49823 assemblies obtained using the sequences from the standard DNA-seq (+3H) and RIMS-seq, compared to the reference genome.

Skin microbiome	Chromosome accession	GC content (%)	Bisulfite-seq motif	RIMS-seq motif	p-value RIMS-seq
Micrococcus luteus	NC_012803.1	73	GGCSGCC	GGCSGCC	7.965455e-572
				CGSNNW	1.74E-154
Propionibacterium acnes	NC_006085.1	60	NA	CSNNNNCG	2.34E-132
Corynebacterium striatum	NZ_CP021252.1	59	GCCGGC	GCCGGC	1.37E-268
				CNNYRNNG	4.72E-135
Acinetobacter johnsonii	NZ_CP010350.1	41	ACGT	ACGT	1.19E-105
				ATCNNGRC	3.12E-123
Streptococcus mitis	NC_013853.1	40	GCNGC	GCNGC	1.540920e-603
			ACGT*	ACGT	6.07E-108
Staphylococcus epidermidis	NC_004461.1	32	NA	NA	NA
**Gut microbiome**	**Chromosome accession**	**GC content (%)**	**Bisulfite-seq motif**	**RIMS-seq motif**	**p-value RIMS-seq**
Bifidobacterium adolescentis	NC_008618.1	59	GATC	GATC	7.609400e-718
			CCNGG	CCNGG	3.970975e-364
Enterobacter cloacae	NC_014121.1	55	CCWGG	CCWGG	3.129282e-1371
Salmonella enterica	NC_003198.1	52	CCWGG	CCWGG	1.071714e-1331
Escherichia coli K12	NC_000913.3	51	CCWGG	CCWGG	2.277395e-1220
Yersinia enterocolitica	NC_008800.1	47	CCWGG	CCWGG	2.314551e-649
Lactobacillus plantarum	NC_004567.2	44	NA	NA	NA
Bacteroides fragilis	NC_006347.1	43	NA	NA	NA
Bacteroides vulgatus	NC_009614.1	42	NA	NA	NA
Helicobacter pylori	NC_000915.1	39	GCGC	GCGC	2.291291e-516
			CYTC*	CCTC	2.387073e-318
Enterococcus faecalis	NC_004668.1	38	NA	NA	NA
Clostridioides difficile	NC_009089.1	29	NA	NA	NA
Fusobacterium nucleatum	NC_003454.1	27	NA	NA	NA

Supplementary Table 3 : Methylases specificity of the synthetic ATCC microbiomes. Motifs followed by an asterisk (*) are motifs for which the bisulfite analysis pipeline had to be adapted to find the motif. For Streptococcus mitis, the ACGT sites were found to be poorly methylated

genome-wide (15% were fully or hemi-methylated). For Helicobacter pylori, literature describes 2 motifs CCTC and TCTTC https://www.ncbi.nlm.nih.gov/pmc/articles/PMC94898/ with an m5C and m4C, respectively. Bisulfite cannot distinguish between m5C and m4C, so it is likely the CYTC motif picked up in the bisulfite data shown in the table results from a composite between the m5C and m4C motifs of H. pylori.
